# A 3D printed capillary holder design for reliable stopped-flow SAX experiments

**DOI:** 10.1107/S1600577525009907

**Published:** 2026-01-01

**Authors:** Paul Wady

**Affiliations:** a Diamond Light Source Diamond House, Harwell Science and Innovation Campus Didcot United Kingdom; Advanced Photon Source, USA

**Keywords:** 3D printing, stop-flow, fluid mixing, experimental technique, sample environments, small angle X-ray scattering (SAXS)

## Abstract

In this paper we discuss adaptations to the BioLogic stopped-flow module (SFM)-400 stop-flow rapid mixing apparatus to enhance reliability for use on a small angle X-ray scattering (SAXS) beamline. Issues with the standard capillary holders are discussed and a 3D printed alternative is presented. The reliability of the new design against leakage is reported. Alternate resins with enhanced solvent tolerance are trialled and discussed.

## Abstract 2

A description of stereolithography printing a disposable, high-reliability alternative to commercially available stopped-flow capillary and cuvette holders is given. The new design offers all the advantages of rapid prototyping for enhanced design flexibility.

## 1. Introduction

Stop-flow is a technique for solution chemistry, allowing observation of reaction kinetics on millisecond time scales. Solutions in syringes are forced though a mixer and into an observation cell. Once a maximum steady velocity has been reached, the ageing time between mixing and observation is at a minimum. At this point the syringes are stopped and a fast-acting valve (the hardstop) at the exit of the observation cell is closed. The mixture then continues to age under the observation technique (often light absorption spectroscopy), and changes to the values as the mixture reacts are recorded. The minimum dead time is therefore limited by maximum flow rate and volume between the mixer and the observation cell.

Stop-flow small-angle X-ray scattering (SAXS) integrates this mixing technique with a synchrotron SAXS beamline. It has been used for observations of protein folding, nanoparticle superlattice assembly, colloids and surfactant self-assembly and self-assembly of organic molecules (Nele *et al.*, 2021 ▸; Angelova *et al.*, 2012 ▸; Fielden *et al.*, 2023 ▸; Grillo, 2009 ▸; Rodriguez-Blanco *et al.*, 2014 ▸).

**Figure 1:**
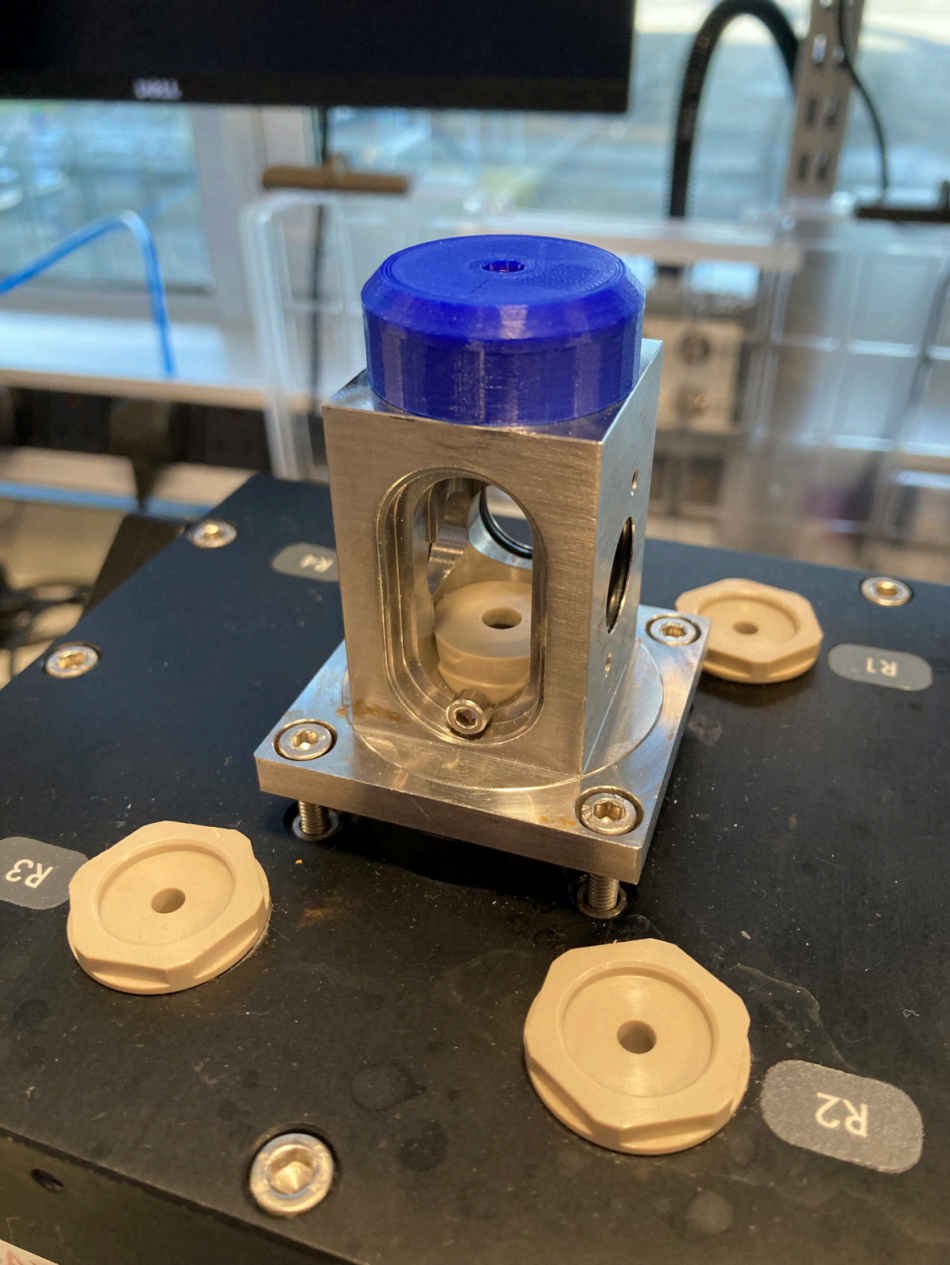
The X-ray head installed on the stop-flow unit. The blue compression screw top forces the tip of the capillary holder (Fig. 2 ▸) inside the head into an o-ring seal in the base of the unit. This compression also pushes a second o-ring seal into the glass capillary. The blue screw top in this image is a 3D printed replacement for the manufacturer’s part which perished due to exposure to solvents. STL files can be found in the supporting information.

At Diamond’s I22 beamline we use a BioLogic (Paris, France) SFM-400 with the manufacturer’s X-ray head attachment and PEEK capillary holders shown in Fig. 1 ▸. This equipment has the capabilities shown in Table 1 ▸.

Synchrotron search procedures impose large delays for any manual intervention, and SAXS data acquisition often requires greater integration time than optical spectroscopy data acquisition. The stepper motor driven BioLogic systems are capable of programmatically performing repeats until the 10 ml syringes have been depleted, while many pneumatically driven stop-flow systems require human intervention before every shot. Each repeat enhances the number of photons exposed at a particular ageing time. The SFM-400 is also capable of successive mixing operations, so reaction conditions may be varied remotely, for example by diluting one of the reactants before mixing it for the final reaction. This implementation of the stop-flow technique is therefore the natural choice for synchrotron research.

## 2. Description of the supplied and printed capillary holders

**Figure 2:**
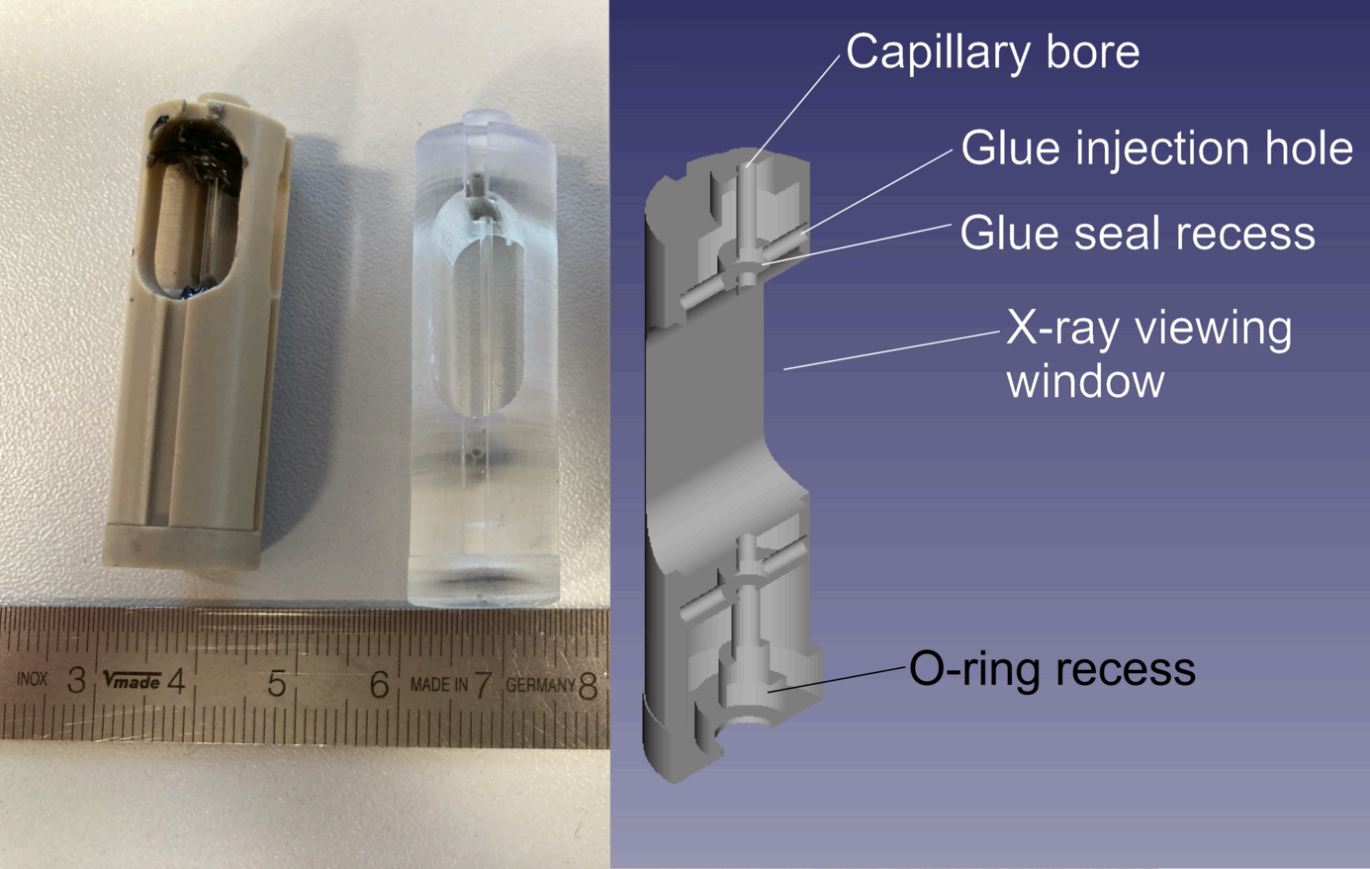
(*a*) The capillary holders discussed in this work: (left) commercially supplied PEEK holder, showing the ep­oxy used to improve sealing; (right) SLA printed resin. (*b*) Cross section through the SLA printed resin capillary holder.

The manufacturer’s capillary holder design [Fig. 2 ▸(*a*), left] seals the capillary to the stop-flow system using a series of o-ring seals. The o-ring at the injection end is compressed by pushing the capillary holder into the X-ray head using the compression screw top, and the o-ring at the exit is compressed by screwing the capillary holder foot into the capillary holder. The effectiveness of these compression seals is limited by variation in the size of the capillary (typically ±0.25 mm) and the capillary holder and features such as the depth of the o-ring recess (which prevents compression beyond the point where further tightening is impossible). In some cases the o-ring is fully recessed, preventing any compression.

Experience shows that the capillary holders tend to leak when operated as-intended with hard FFKM o-rings and we have therefore developed a practice of backing the o-ring seals with ep­oxy [see Fig. 2 ▸(*a*), left] or stacking multiple o-rings. This has two drawbacks: (i) the ep­oxy blocks the beam and contributes to scattering; to avoid this the beam must pass through the capillary higher up, thus increasing the volume between the mixer and observation and increasing dead time; (ii) when the capillary breaks, there must be a time-consuming ungluing process in which the capillary holder is soaked in chloro­form for a few days to remove the ep­oxy before reuse can be considered.

We have therefore designed a 3D printed, disposable capillary holder which provides excellent sealing [Fig. 2 ▸(*a*), right, and Fig. 2 ▸(*b*)]. This design is easily modified to allow larger and smaller diameter capillaries, as might be required to minimize sample volumes or increase scattering thickness (see the supporting information for STL files for capillaries with 1, 1.5 and 2 mm outer diameters).

**Figure 3:**
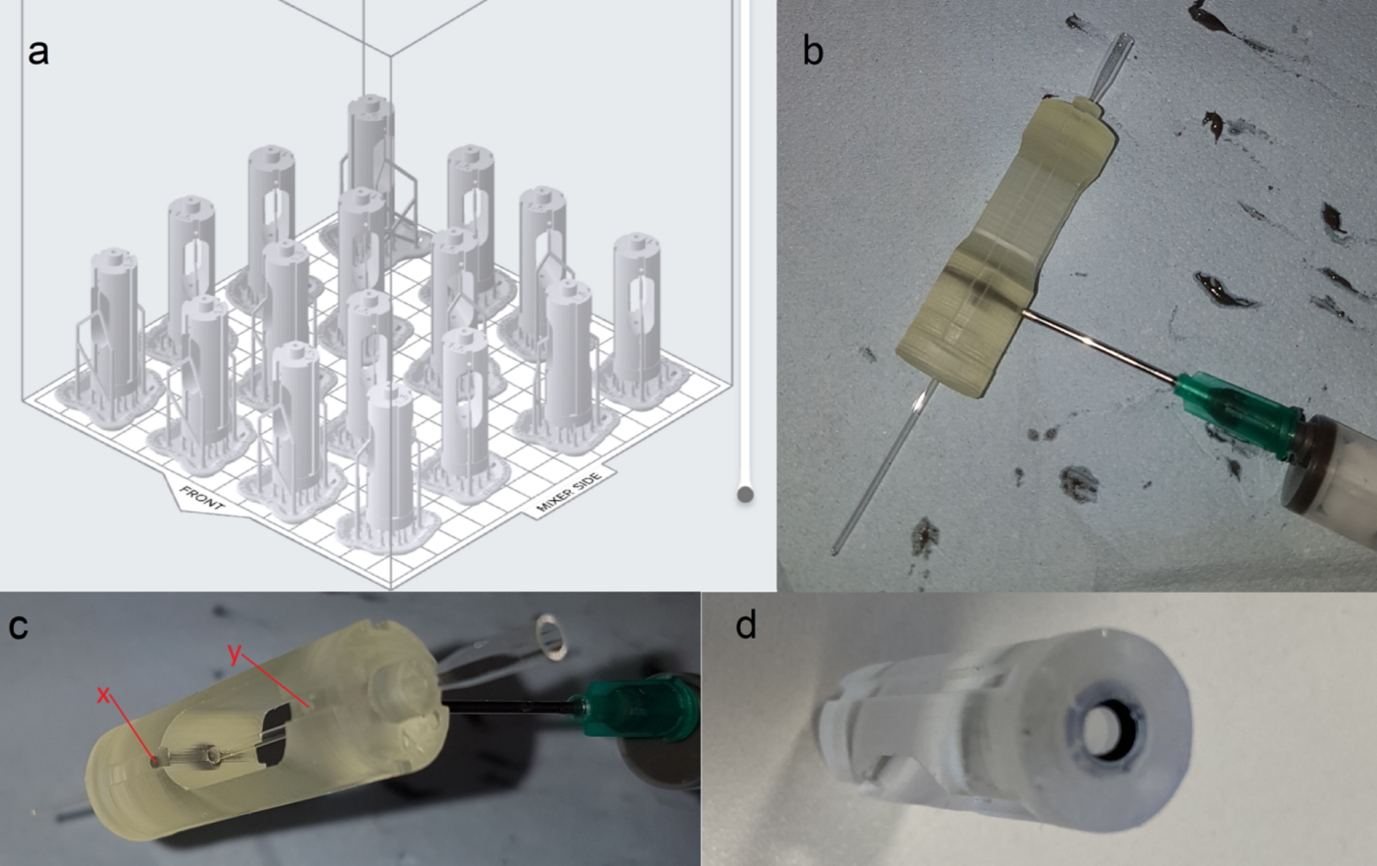
(*a*) Capillary holder printing set up. (*b*) Glue injection. (*c*) Note how the lower channel, labelled ‘x’, can be seen to be filled with ep­oxy, but that the upper channel, labelled ‘y’, is yet to be injected. (*d*) O-ring exit seal.

The design is printed vertically, allowing up to 16 units to be produced in a single 9 h print run with a Formlabs Form 2 printer [Fig. 3 ▸(*a*)] without overlapping the support rafts (which makes print removal difficult). This is followed by sonicating to remove excess resin (∼20 min), drying and final UV cure (∼30 min).

**Figure 4:**
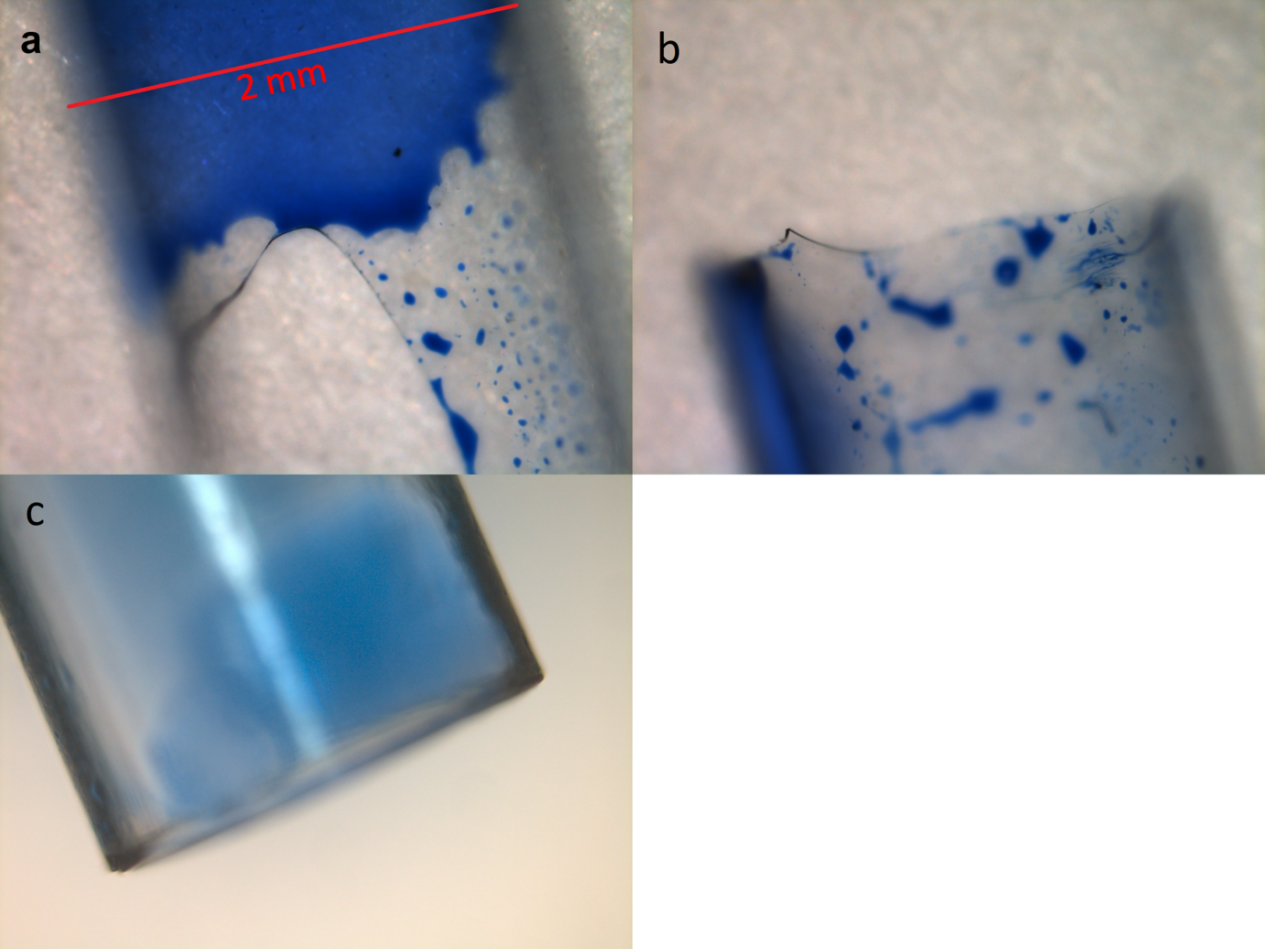
(*a*) Snapped glass capillary end, (*b*) scored glass capillary end and (*c*) cut PC capillary. Each capillary has a nominal diameter of 2 mm and has been dipped in ink to enhance visibility.

Quartz glass capillary tubes (Capillary Tube Supplies Ltd, Bodmin, UK) were cut to length using an alumina scoring tool (Trajan Scientific and Medical Ltd), and polycarbonate tubes were cut with a razor blade to produce squared-off ends; snapping glass capillaries creates ragged edges and fractures that may allow leaks around the seal [Figs. 4 ▸(*a*)–4(*c*)]. The capillary is inserted in the holder and ep­oxy is injected into the glue recess as shown in Figs. 3 ▸(*b*) and 3(*c*). The flow of the ep­oxy compensates for much larger gaps between capillary and holder than is possible for o-rings. The capillary holder is then left overnight to allow the ep­oxy to dry, and finally the bottom o-ring [Fig. 3 ▸(*d*)] is inserted to seal to the hard stop. With this design we have had extremely reliable operation, see Table 2 ▸.

## 3. Chemical resistance

Printing this design using stereolithography (SLA) printer technology limits our production to resin materials. It has worked well with Formlabs Clear v4, and Formlabs Rigid 10K resins. Of the two, Rigid 10K is reported to have the better chemical resistance (Formlabs, 2022 ▸). This resistance will still be severely limited compared with the PEEK originals, limiting the experiments for which the capillary holder is usable. The ep­oxy seal is also significantly less resistant to chemical attack than FFKM o-rings.

Experiments using 9:1 (v/v) water:tetra­hydro­furan­(thf) mixture with a 45 min ageing time used Clear v4 resin cartridges with no problems. Follow-up tests soaking a resin cartridge in 100% thf for 1 h resulted in a 1% mass increase, with no substantial change in hardness or dimensions.

## Supplementary Material

STL file for 1 mm capillary holder. DOI: 10.1107/S1600577525009907/vy5045sup1.bin

STL file for 1.5 mm capillary holder. DOI: 10.1107/S1600577525009907/vy5045sup2.bin

STL file for 2 mm capillary holder. DOI: 10.1107/S1600577525009907/vy5045sup3.bin

STL file for X-ray head screw top. DOI: 10.1107/S1600577525009907/vy5045sup4.bin

## Figures and Tables

**Table 1:** Basic characteristics of the SFM-400 (SFM, undated ▸)

Characteristic	Value
Number of syringes	4
Syringe size	10 ml
Flow rate	1–8 ml s^−1^
Minimum dead time	0.8 ms
Minimum volume per shot	400 µl

**Table 2:** Reliability of capillary holders (ten of each design were tested)

	Quartz glass special tubing			Polycarbonate
Diameter (mm)	2	1.5	1	2
Survived 20 shots of 5 m µs^−1^	6	3	8	10
Leaked or burst in <20 shots of 5 ml µs^−1^	3	6	2	0
